# Supramolecular Solvent-Based Microextraction of Selected Anticonvulsant and Nonsteroidal Anti-Inflammatory Drugs from Sediment Samples

**DOI:** 10.3390/molecules25235671

**Published:** 2020-12-01

**Authors:** Sylwia Bajkacz, Paulina Adamczewska, Klaudia Kokoszka, Elżbieta Kycia-Słocka, Adam Sochacki, Ewa Felis

**Affiliations:** 1 Department of Inorganic Chemistry, Analytical Chemistry and Electrochemistry, Faculty of Chemistry, Silesian University of Technology, B. Krzywoustego 6 St., 44-100 Gliwice, Poland; pooooolina@wp.pl (P.A.); klaudia.kokoszka@polsl.pl (K.K.); e.slocka@interia.pl (E.K.-S.); 2 Environmental Biotechnology Department, Faculty of Power and Environmental Engineering, Silesian University of Technology, Akademicka 2 St., 44-100 Gliwice, Poland; adam.sochacki@polsl.pl (A.S.); ewa.felis@polsl.pl (E.F.); 3 Department of Applied Ecology, Faculty of Environmental Sciences, Czech University of Life Sciences Prague, Kamýcká 129, 165 00 Prague, Czech Republic

**Keywords:** supramolecular solvent-based microextraction, Speedisk, micropollutants, liquid chromatography

## Abstract

The increase in the production and consumption of pharmaceuticals increases their presence in the global environment, which may result in direct threats to living organisms. For this reason, there is a need for new methods to analyze drugs in environmental samples. Here, a new procedure for separating and determining selected drugs (diclofenac, ibuprofen, and carbamazepine) from bottom sediment and water samples was developed. Drugs were determined by ultra-high performance liquid chromatography coupled with an ultraviolet detector (UHPLC-UV). In this work, a universal and single-step sample treatment, based on supramolecular solvents (SUPRAS), was proposed to isolate selected anticonvulsants and nonsteroidal anti-inflammatory drugs (NSAIDs) from sediment samples. The following parameters were experimentally selected: composition of the supramolecular solvent (composition THF:H_2_O (*v*/*v*), amount of decanoic acid), volume of extractant, sample mass, extraction time, centrifugation time, and centrifugation speed. Finally, the developed procedure was validated. A Speedisk procedure was also developed to extract selected drugs from water samples. The recovery of analytes using the SUPRAS procedure was in the range of 88.8–115%, while the recoveries of the Speedisk solid-phase extraction procedure ranged from 81.0–106%. The effectiveness of the sorption of the tested drugs by sediment was also examined.

## 1. Introduction

Due to the progressive development of medicine, an increasing number of drugs have been produced throughout the world. Along with an increase in production levels, the scale of consumption and availability of drugs has also increased because more and more medicinal substances are available as over-the-counter (OTC) drugs. Even after ingestion, most drugs do not lose their biological activity, which means that in unmetabolized form or as active metabolites, they are released into the environment. In European countries, about 4000 different biologically-active compounds may enter the environment [1]. Drugs, despite their high chemical stability, can undergo various chemical and physical transformations [2], including direct photodegradation—caused by the absorption of light or indirect photodegradation–to form radicals [3]. The effects caused by the presence of pharmaceutical residues in the environment have resulted in the development of related scientific research. Since 1990, the number of publications and research related to this subject has exponentially increased due to the increasing use of drugs and the development of sensitive and selective analytical techniques that allow the detection of trace amounts of chemical compounds in complex matrices of environmental samples [4].

Until now, only a small portion of pharmaceuticals in environmental samples has been identified, and the research has mostly focused on their occurrence, fate, and elimination from sewage treatment plants. Most published research concerns aquatic environmental pollution by nonsteroidal anti-inflammatory drugs (NSAIDs) and antibiotics. NSAIDs are the most commonly prescribed drugs in the world, and they include diclofenac and ibuprofen, which have anti-inflammatory, antipyretic, and analgesic effects. In many countries, they are also available as OTC drugs and sold not only in pharmacies, but also at gas stations, cosmetic stores, grocery stores, and other places. The absorption of these drugs by the gastrointestinal tract occurs very quickly and is excreted from the body in up to 24 h [5].

In the environment, antiepileptic drugs are also widely distributed. The most frequently identified representative of this group of drugs is carbamazepine [1], which has psychotropic, neurotropic, and anticonvulsive effects. After administration, it is absorbed almost completely from the gastrointestinal tract, and 70–80% is bound to plasma proteins. It penetrates the blood-brain barrier, the placenta, and reaching the female food. The half-life of carbamazepine in the body during the initial period of use is 72 h [6].

Table S1 (Supplementary Material) presents the structural formulas of diclofenac, ibuprofen, carbamazepine, and acenocoumarol (used as an internal standard), and selected properties of the drugs.

Pharmaceuticals can be introduced into the environment in many ways. Their low concentrations can be explained by a large dilution (up to the level of ng L^−1^) and adsorption on suspended solids and bottom sediments [7]. Due to the increasing attention devoted to medicines entering the environment, there is a need to develop new methods for determining them in environmental samples, followed by selective analysis with the lowest possible limit of detection (LOD) and limit of quantification (LOQ).

The most common techniques for the preparation of environmental samples for the determination of nonsteroidal anti-inflammatory drugs are solid-phase extraction (SPE) and solid-phase microextraction (SPME) [8]. Sample preparation methods for soils and bottom sediments for NSAID assays are based on multistage procedures involving lyophilization and the use of solid-liquid extraction assisted with microwave radiation (MAE) and ultrasound (UAE) or accelerated solvent extraction (ASE) [9,10,11,12,13,14,15,16,17,18]. Alternative extraction methods are being developed. One of them is microextraction using supramolecular solvents—which can be used to isolate organic compounds with different polarities from environmental samples [19]. Nanostructured liquids called supramolecular solvents (SUPRAS) are liquids that are rich in surfactants. Supramolecular solvents are spontaneously formed from colloidal solutions of amphiphilic compounds due to the sequential self-assembly of micelles and coacervation. The composition of nanostructured liquids greatly impacts the extraction efficiency of organic compounds, the concentration factor (CF), and the suitability for dissolving analytes with a wide range of polarities. 

Procedures to produce SUPRAS solvents compatible with chromatographic and electrophoretic techniques are currently being developing [20]. Very often, supramolecular solvent-based microextraction (SUPRAS microextraction) uses decanoic acid (DeA) with tetrahydrofuran (THF) as the amphiphilic compound. To obtain the best concentration factors, an appropriate volume of SUPRAS and amount of decanoic acid and THF should be selected [20]. It is also necessary to choose the appropriate extraction temperature, the addition of salts, bases, acids, alcohols, polymers, and other organic compounds [21,22]. SUPRAS microextraction has been used to separate analytes from solid and liquid samples. In the bottom sediment estrone, 17β-estradiol, estriol, 17α-ethynyl-estradiol, and bisphenol A were extracted using SUPRAS [23], with a detection limit of 0.03–0.40 ng g^−1^. The stereoisomers of mecoprop and dichloroprop were isolated from the soil (*LOD* = 0.03–0.10 ng g^−1^) [24]. SUPRAS microextraction has been used to extract herbicides [25,26], mecoprop stereoisomers [27], phthalates [28], and ethinyl estradiol [29] from water.

The aim of this study was to develop new procedures for the isolation of selected drugs belonging to various therapeutic groups, i.e., diclofenac, ibuprofen (NSAIDs), and carbamazepine (anticonvulsant) from bottom sediment and water samples and a determination method using UHPLC-UV. The SUPRAS microextraction parameters were selected for the extraction of drugs from the bottom sediment, and the Speedisk procedure was developed to separate drugs from water samples. The study also estimated the sorption capacity of the sediment towards selected drugs.

## 2. Results and Discussion

### 2.1. Development of Chromatographic Conditions

In this study, the procedure for the simultaneous determination of three drugs (carbamazepine, ibuprofen, and diclofenac) using a UHPLC-UV method was reported. Selected analytes were carefully selected. Carbamazepine is a compound that is not eliminated during wastewater treatment, and its concentration in treated wastewater is often at the same level or even higher than in raw wastewater due to its accumulation in the treatment system. Diclofenac is only removed to a small extent in wastewater treatment plants, while ibuprofen is found in sewage sludge in higher concentrations than other substances [30].

Chromatographic conditions were developed based on the lipophilicity of compounds (log*P*), solubility in water and organic solvents, and *pK*_a_ values (Table S1). Selected drugs were analyzed using reversed-phase UHPLC, which has many advantages, such as a short analysis time, low consumption of expensive and harmful organic solvents, and increased sensitivity and resolution. The analysis time was 5.5 min. In most cases, drugs were determined using high-performance liquid chromatography, which prolonged the analysis time. The stationary phase was a Poroshell 120 EC-C18 column. Earlier literature does not describe the use of a core-shell column the determination of drugs in sediment or soil samples. The stationary phase particles had a diameter 2.7 μm and consisted of a nonporous core covered with a porous coating, which enabled a high efficiency and lower backpressure.

During the development of the chromatographic system, the flow rate and the composition of the mobile phase were changed. Table 5 shows the applied gradient program, which started with a high proportion of 0.05% trifluoroacetic acid in water. As time passed, the amount of organic modifier—acetonitrile in the mobile phase it increased. This increased the elution power and eliminated the most nonpolar compounds from the chromatography column. The temperature of the column was 20 °C, and the injection volume was 3 μL. A spectrophotometric detector was used for determinations. The selected analytical wavelength (λ = 221 nm) was close to the maximum absorption of the analyzed compounds. To calibrate the method, the internal standard method was used. Acenocoumarol was chosen as the internal standard because the maximum absorption of this compound (λ = 205 nm) was close to the analytical wavelengths of diclofenac, ibuprofen, and carbamazepine. In addition, its properties and retention time (*t*_R_ = 4.80 min) are similar to the analyzed drugs. In the chromatogram, the signal from acenocoumarol does not overlap with any signal of the matrix, and the compound does not interfere with the drugs being measured.

**Figure 1:**
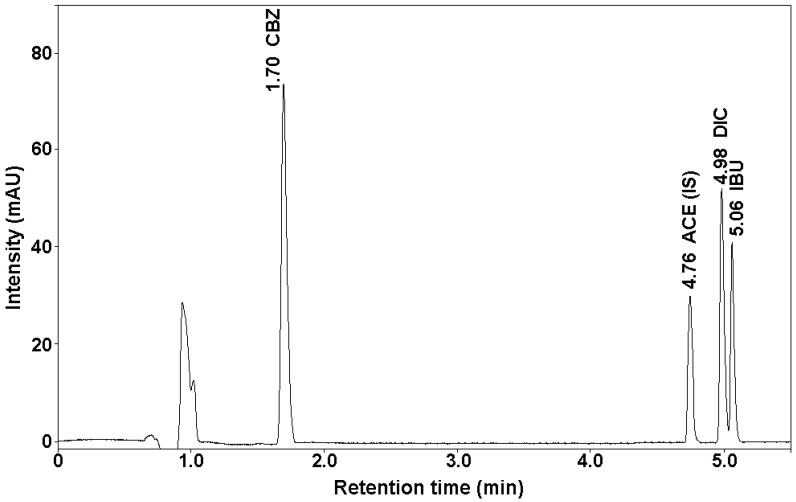
Chromatogram of analytes standard solution and internal standard.

Below (Figure 1) is an example of a chromatogram of analyzed drugs and an internal standard obtained under the chromatographic conditions.

### 2.2. SUPRAS Microextraction

The extraction procedure of three of the most commonly encountered drugs in the environment from bottom sediment samples was developed. The procedure began with drying the collected sample. This treatment is particularly important, since residual water impedes the penetration of the sample by the organic solvent. Access to adsorbed analyte is difficult when there is water in the matrix, which reduces its solubility in organic extractants [31]. After drying at room temperature, the precipitate was ground in a mortar, sieved, and enriched. As part of the research, the influence of various parameters on the effectiveness of drug separation from bottom sediment samples was determined. The initial conditions were as following: 0.3 g of the bottom sediment sample was weighed and enriched so that the final concentration of drugs in the sample was 5 μg mL^−1^. The analytes were then extracted using 1000 μL supramolecular solvent consisting of 6.5 g decanoic acid and THF:H_2_O = 30:70 (*v*/*v*) during 30 min. The separation of the precipitate from the extract was performed by centrifugation at 7000 rpm for 5 min. During optimization, one parameter was changed, the others remained constant as described above. The effectiveness of SUPRAS extraction was evaluated based on the drug recovery (expressed as a percentage). The amount of solvent used for the extraction, the time of the procedure, and the value of the enrichment factor were also taken into account.

#### 2.2.1. Composition of Supramolecular Solvent

**Figure 2:**
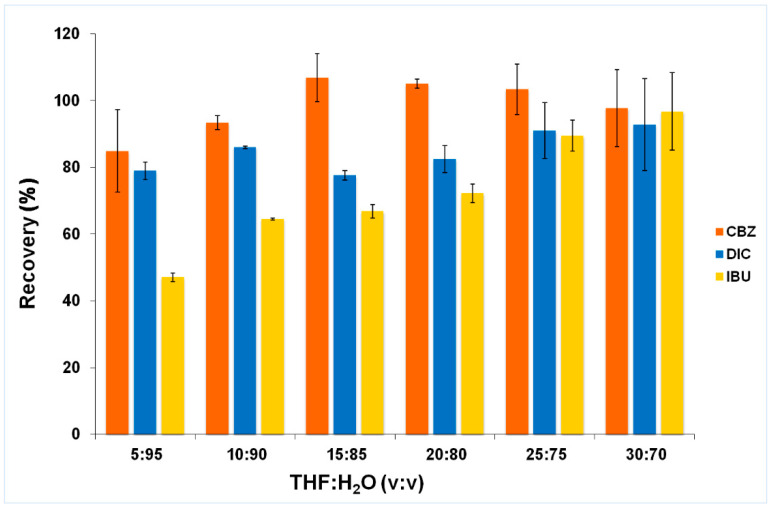
Effect of the THF:H_2_O volume ratio on analyte recovery.

In the first stage, the composition of the supramolecular solvent was compared. For this purpose, six solutions with different volume ratios of THF:H_2_O were prepared. The best results were obtained using an extractant with a composition of THF:H_2_O = 30:70 (*v*/*v*), which was used for further experiments. During chromatographic analysis, an increase in the intensity of the signals of the supramolecular solvent, along with a decrease in the tetrahydrofuran fraction, were observed. Using the smallest amount of THF solvent, these signals overlapped with the analyte peaks. Figure 2 shows the influence of the volume ratio of THF:H_2_O on the analyte recovery.

The effect of the amount of decanoic acid on the SUPRAS microextraction efficiency was also investigated. Extractants containing 1.0, 3.0, 6.5, and 10 g of decanoic acid were tested. The high ability of reverse decanoate micelles to extract drugs is associated with the formation of hydrogen bonds and hydrophobic interactions between the surfactant and the analyzed compounds. Based on the results, the amount of DeA does not significantly affect the extraction efficiency. Further studies were performed using a supramolecular solvent prepared with 6.5 g DeA because the extraction efficiency was the highest. Figure S1 in the Supplemental Material shows the effect of the amount of decanoic acid on the recovery of analytes.

**Figure 3:**
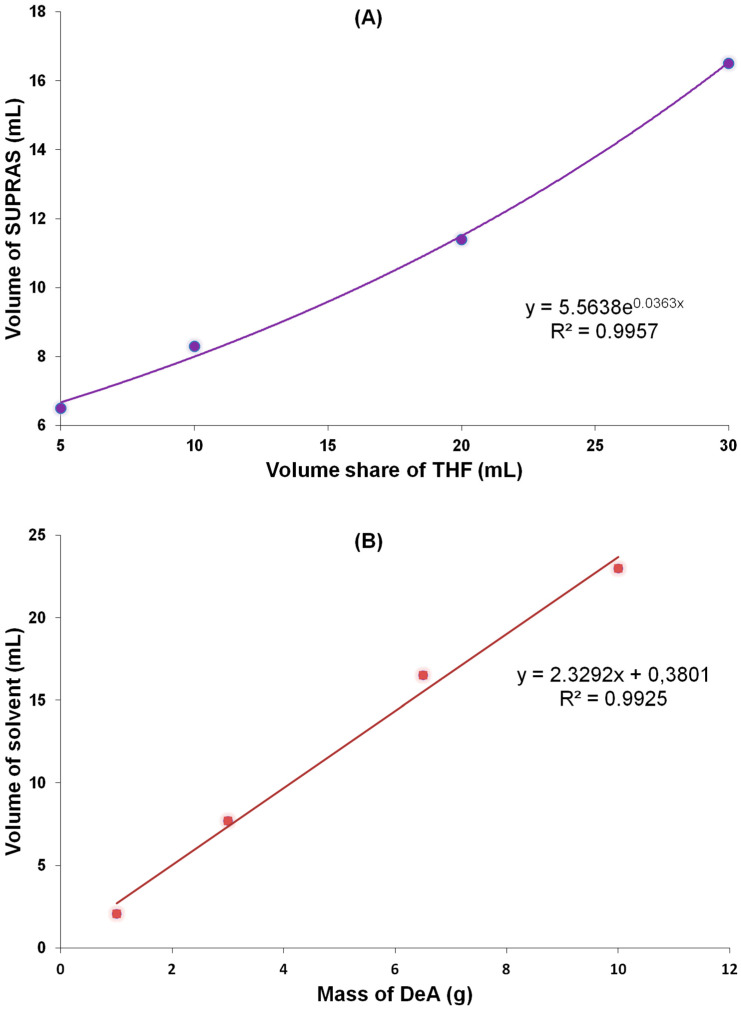
Effect of (**A**) volume of THF and (**B**) mass of decanoic acid per volume of supramolecular solvent.

The volume of the resulting extractant was affected by pH, the amount of carboxylic acid used, the length of the alkyl chain of the acid, and the THF:H_2_O volume ratio. As part of this work, the effect of decanoic acid and the THF:H_2_O volume ratio on the volume of the supramolecular solvent produced was examined (Figure 3). THF has a low Hildebrand solubility parameter (δ). Solvents with the lowest value of this parameter have the highest solubility of carboxylic acids. Carboxylic acids with the shortest alkyl chains are typically used to produce SUPRAS, due to the small amount of THF needed for coacervation [32]. The graphs show that the volume of supramolecular solvent produced depends linearly on the amount of decanoic acid (solvent composition remains unchanged [33]) and increases exponentially with the increase of THF in SUPRAS, which is in accordance with the literature [32,33]. Decanoic micelles became increasingly dilute within increasing THF content in the supramolecular solvent. Together with the increase in the volume fraction of THF in SUPRAS, a reduction in the intensity of signals from DeA was observed in the obtained chromatograms. This means that the composition and properties of SUPRAS depend on the volume ratio of THF:H_2_O and the weight of decanoic acid only affects the volume of the solvent prepared.

#### 2.2.2. Volume of the Supramolecular Solvent

In the next stage, the volume of the supramolecular solvent used for the extraction was selected. The goal was to obtain a good recovery while obtaining the largest enrichment ratio (EF). The extractant volume was checked from 400 to 1600 μL. The best results, low standard deviations, and high recovery of the studied drugs were obtained when using more than 400 μL supramolecular solvent. A volume of 400 μL of extractant was not sufficient to penetrate the sample and separate the analyte. A volume of 700 μL was selected for further analysis. When analyzing the samples extracted with this amount of solvent, a high enrichment factor, recovery from 95% to 102%, and a standard deviation from 1.2 to 2.0% were obtained. Figure S2 shows the effect of the volume of the extractant on the recovery of the tested drugs and the value of the enrichment factor.

#### 2.2.3. Sample Weight

The optimum mass of the bottom sediment sample was selected. Before extraction, the sludge weights with masses from 0.1 to 0.5 g were enriched with analytes. The greatest enrichment factor was obtained. For 0.5 g, the volume of extractant was too small to sufficiently penetrate the sample, and a lower recovery was obtained (75.7–86.9%). After drug extraction from the bottom sediments with weights 0.1 g and 0.2 g, the obtained results were not sufficiently repeatable, as indicated by the high standard deviations (9.1–13.9%). For further studies, a weight of 0.4 g was used, which obtained the largest recovery and a high enrichment factor. The enrichment factor increased linearly with increasing sample mass. The obtained results are shown in Figure S3.

#### 2.2.4. Extraction Time

The advantage of microextraction with a supramolecular solvent is a short sample preparation time for analysis compared with other time-consuming extraction techniques. The extraction time was selected to define how long was required for mass transfer of the analyte from the sample to the solvent. The analyte transfer largely depended on the diffusion rate resulting from the degree of fragmentation and sample viscosity [34]. Samples were analyzed that had been extracted between 5 and 40 min. After applying a 20-min extraction, during which equilibrium was established, the recovery of the studied drugs was in the range of 101–108%. Further extending the extraction time did not increase the extraction efficiency; therefore, extraction was carried out for 20 min. Figure S4 shows the effect of the extraction time on the recovery of the analyzed compounds.

#### 2.2.5. Centrifugation Time and Speed

The sample was centrifuged during sample preparation to separate solid particles from the extractant. It was determined how centrifugation time affected the recovery of analytes, and the results are shown in Figure S5a. A good separation of solid particles from liquid and high analyte recovery was obtained after 10 min of centrifugation.

The last of the selected parameters were the centrifugation rotation, which did not significantly affect the extraction efficiency; therefore, a centrifugation speed of 9000 rpm was selected to better separate the precipitate from the supramolecular solvent. Figure S5b shows the effect of centrifugation speed on the recovery of the analyzed drugs.

#### 2.2.6. SUPRAS Microextraction Procedure

**Figure 4:**
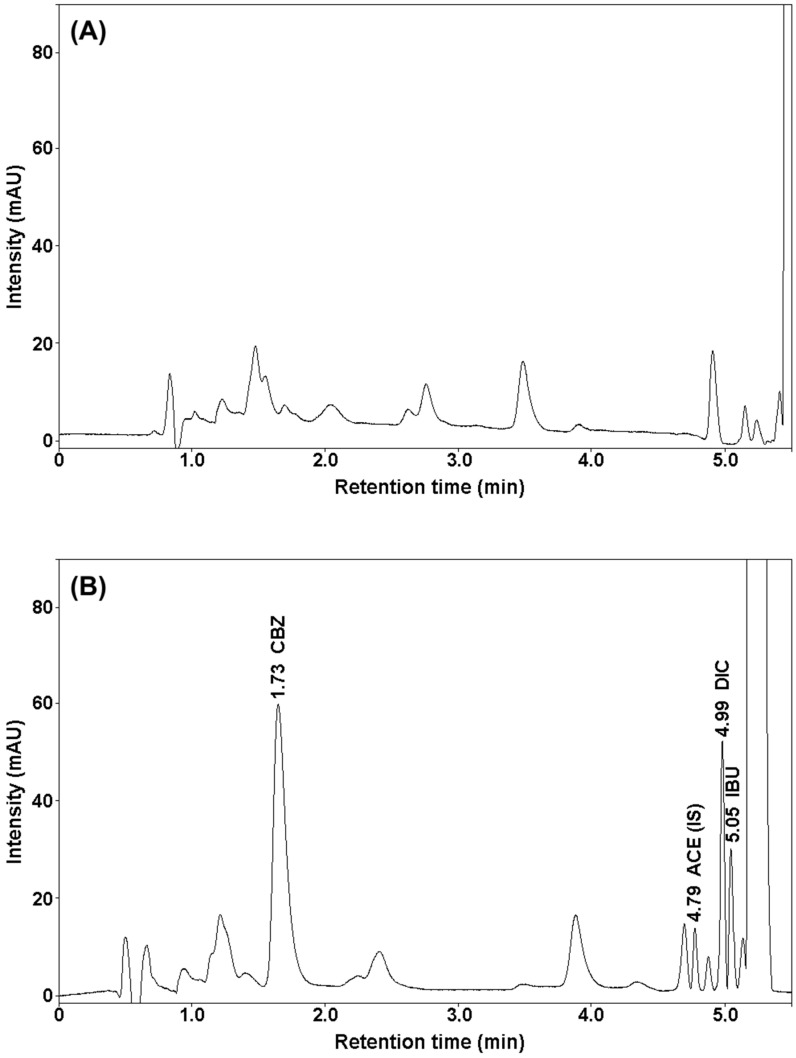
Chromatograms of bottom sediment sample extracts: (**A**) without added standards (blank sample), (**B**) spiked with drugs obtained after SUPRAS microextraction.

Based on the obtained results, a new SUPRAS microextraction procedure was developed to extract carbamazepine, diclofenac, and ibuprofen from bottom sediment samples. SUPRAS are multi-target solvents made up of self-assembled amphiphiles. They offer multiple extraction interactions (dispersion, polar, hydrophobic, etc.) and are excellent candidates to develop generic and fast sample treatment procedures at low-cost. A high enrichment factor and good extraction efficiency were obtained using a 0.4 g bottom sediment sample. The analytes were then extracted using 700 μL supramolecular solvent consisting of 6.5 g decanoic acid and THF:H_2_O = 30:70 (*v*/*v*). A high recovery of analytes at low standard deviations was obtained after only 20 min of extraction. The exact separation of the precipitate from the extract and the high extraction efficiency was achieved by centrifugation at 9000 rpm for 10 min. Table 1 presents the recovery of analytes after applying the developed extraction procedure. The analyses were carried out three times for sediment samples enriched with selected drugs at three concentrations: 1.25, 10, and 20 μg g^−1^. The recovery of the internal standard after applying the developed procedure was 92.6%. Figure 4 shows a chromatogram of bottom sediment extract spiked with CBZ, IBU, DIC (20 μg g^−1^), and ACE (10 μg g^−1^) and a blank chromatogram (bottom sediment extract without added test drug standards).

### 2.3. Speedisk Solid-Phase Extraction

Speedisk solid-phase extraction was used to extract selected drugs from water samples. The filling of the extraction discs used was silica gel modified with octadecyl groups. The recovery of analytes ranged from 81% to 106% (Table 1) and IS recovery after the procedure was 77%. The advantage of the Speedisk extraction is a high enrichment factor, which influences the analyte determination efficiency [31]. This is particularly important in the case of the separation of pharmaceuticals from water samples, due to their low concentrations in real samples. The enrichment factor for the Speedisk procedure was *EF* = 1000. The disadvantage of this method is the relatively high amounts of organic solvents used for conditioning and elution.

**Figure 5:**
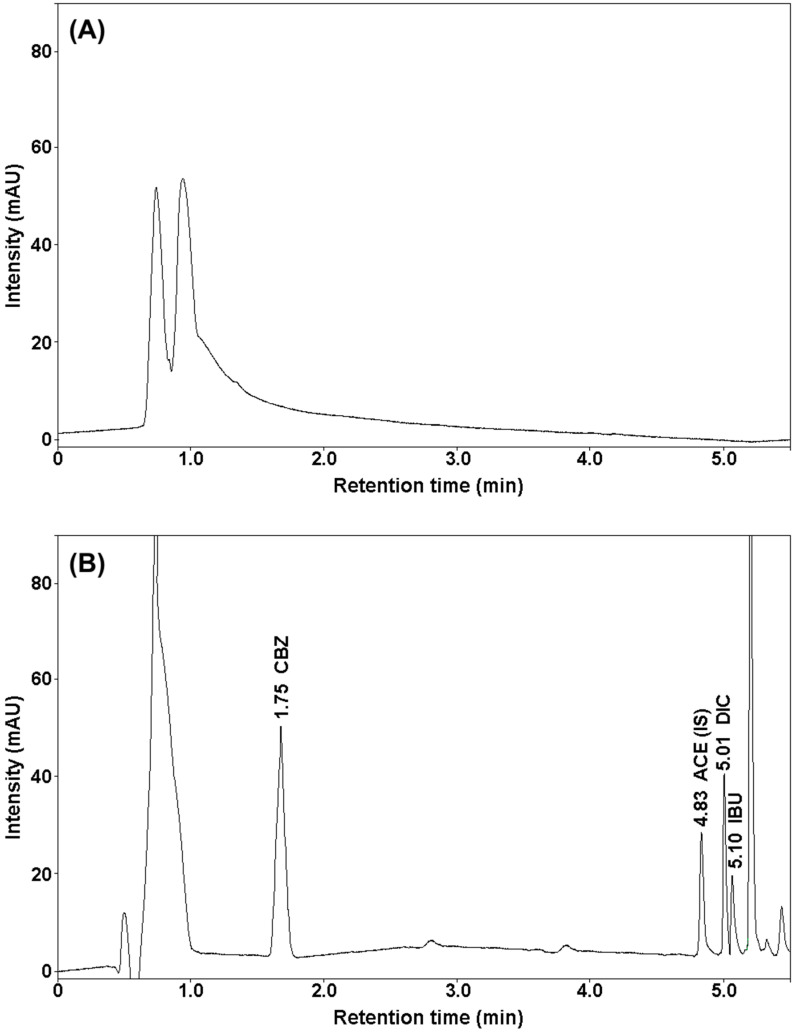
Chromatograms of water sample extracts: (**A**) blank sample, (**B**) samples of water spiked with drugs obtained after Speedisk solid-phase extraction.

Figure 5 shows the chromatogram of water extracts (spiked with CBZ, IBU, DIC) and the water sample chromatogram without added drug standards.

### 2.4. Method Validation

The developed method has been validated. Calibration curves and the range of linearity, detection limits (LOD) and quantification (LOQ), extraction recovery, precision, and accuracy of the method were determined.

#### 2.4.1. Linearity and Sensitivity

The first stage of validation was obtaining the calibration curves. Solutions of the drugs CBZ, DIC, and IBU were prepared in the concentration range from 0.5 to 25.0 μg g^−1^ for the SUPRAS extraction and 0.5–5.0 μg L^−1^ for the Speedisk extraction. Each time, an internal standard was added to eliminate the influence of apparatus factors and the analyst (human factor) on the measurement result. For each curve, the equation and the correlation coefficient (*R*^2^) (Table 2) were determined. In the selected concentration range, the obtained curves were linear. The sensitivity of the method was determined by the limit of detection (LOD) and limit of quantification (LOQ). The lowest concentration of the compound for which the relative error was lower than 20% was taken as the LOQ. The LOD was obtained by dividing the LOQ by 3. The limit of quantification for all drugs tested was 1.25 μg g^−1^, and the detection limit was 0.42 μg g^−1^ for the bottom sediment sample after applying the SUPRAS method.

#### 2.4.2. Accuracy and Precision

To evaluate the accuracy and precision of the method, a series of three measurements was performed for three analyte concentration levels in the range of the standard curve. QC samples were prepared as described in the experimental section. The mean drug concentration, relative error (RE), standard deviation (SD), relative standard deviation (RSD), and coefficient of variation (CV) were calculated. The obtained results are shown in Table S2. In all cases, the coefficient of variation was lower than 15%, demonstrating the good precision of the method. Low relative errors (RE < 15%) indicate that both methods are accurate. It can also be seen that in the case of Speedisk extraction, all parameters were lower than those of SUPRAS extraction, which is related to the lower impact of the matrix on the measurement result.

### 2.5. Comparison of Proposed SUPRAS-UHPLC-UV and Speedisk-UHPLC-UV Methods with Other Reported Methods

Table 3 and Table 4 compare the developed SUPRAS method and the Speedisk method with other methods described in the literature for analyzing drugs in sediment and water samples.

### 2.6. Study of the Sorption Efficiency of Selected Drugs on Soil

The sorption efficiency of carbamazepine, diclofenac, and ibuprofen on soil was investigated. The sorption effectiveness was evaluated by analyzing three parallel samples, prepared with eight different drug concentrations. Only ibuprofen adsorbed on the sediment (no sorption was found on the walls of the vessel). To confirm the sorption of ibuprofen, the developed SUPRAS microextraction procedure was used. Consequently, ibuprofen was found in soil extracts. Figure S6 shows the obtained Freundlich adsorption isotherm. The solid-liquid partition coefficient *K*_D_ for ibuprofen was 2.64 L kg^−1^ (Figure S7).

Sorption depends on the size of chemical compounds, their lipophilicity, and surface charge [45]. The sorbent parameters (soil), such as the content of organic carbon (OC) and inorganic compounds, soil pH, cation exchange capacity, and grain size also have a significant impact on the sorption efficiency [46]. Ibuprofen and diclofenac sorption is strongly correlated with soil pH and the organic carbon content in soil and the temperature [47] For a pH lower than the *pK*_a_ of ibuprofen (4.85) or diclofenac (4.0) (Table S1), the molecules are undissociated, while at pH values higher than their *pK*_a_, these compounds are present in their anionic form [46,48]. The typical pH of soil in Poland is within the range of 4.5–5.5 [49], and this pH range assumed for the tested soil. The adsorption in a batch sorption test is governed by the pH of the soil suspension. Within the pH range of 4.5–5.5, approx. 70% to 20% of ibuprofen, and only 20% to 2% of diclofenac, occurs in their undissociated form. The anionic forms of ibuprofen and diclofenac have much higher affinity for the water phase than for the soil. This may cause ibuprofen and diclofenac anionic forms to be repelled from the soil surface [50]. Because of the higher proportion of the undissociated form of ibuprofen as compared with diclofenac, ibuprofen was adsorbed to a considerably higher extent than diclofenac. Carbamazepine exists in its undissociated form within a wide range of pH including the experimental range of 4.5–5.5, but unlike for ibuprofen its adsorption was negligible. This could be associated with different sorption mechanisms that have been reported for those drugs [51]. The low adsorption affinity of carbamazepine could have been also due to its high molecular weight, neutral charge, and high resistance to hydrolysis in natural conditions, which prevent it from strongly interacting with the soil matrix [52].

In the literature, the sorption of ibuprofen under natural conditions in environmental samples is described as weak or medium. In studies conducted on coastal soils, the value of *K*_D_ ranged from 0.66–1.26 L kg^−1^ for soils with a high OC content and 0.63 L kg^−1^ for sandy soils with a low OC [46]. In soils collected from an agricultural field and park, the organic carbon content was in the range of 0.9–1.1%, and *K*_D_ was 1.00–7.00 L kg^−1^, so it was slightly higher than for coastal soils [53]. The research carried out on soils from wetlands under aerobic and anaerobic conditions confirmed the high mobility of ibuprofen in aquatic environments due to its low sorption to the soil (*K*_D_ 0.08–2.62 L kg^−1^) [54]. The *K*_D_ value determined for ibuprofen in this study was within the range mentioned in the literature.

In studies of pharmaceuticals adsorption in river sediment samples, it was shown that the sorption of carbamazepine and diclofenac was higher than for ibuprofen, namely KD for CBZ was in the range of 5.57–24.55 L kg^−1^ and KD for DIC was 4.04–33.01 L kg^−1^ [55]. Similar results were also obtained for soil samples collected from dam reservoirs, where KD of CBZ was 2.9–5.5 L kg^-1^, and 4.8–5.5 L kg^−1^ for DIC [20], and in tropical soil samples CBZ was 0.83–3.38 L kg^−1^ [56]. 

## 3. Materials and Methods

### 3.1. Standards, Chemicals, and Materials

Analytical standards of carbamazepine (CBZ) (≥99% purity), ibuprofen (IBU) (≥99% purity), and diclofenac (DIC) (≥98.5% purity) were purchased from Sigma-Aldrich Chemie (Steinheim, Germany). Internal standard (IS) of acenocoumarol (ACE) were purchased from U.S. Pharmacopeial Convention (Rockville, MD, USA). HPLC-grade acetonitrile (ACN), water, and trifluoroacetic acid (TFA) were obtained from Merck (Darmstadt, Germany). Analytical-grade tetrahydrofuran (THF), methanol, acetone, hydrochloric acid solution 35–38%, sulfuric acid (VI) 98%, and decanoic acid 98% (DeA) were purchased from POCH S.A. (Gliwice, Poland), CHEMPUR (Piekary Śląskie, Poland) and Sigma-Aldrich Chemie.

A sample of bottom sediment was collected at the shore of Lake Czechowice in Gliwice at a depth of 30 cm on 26 October 2014 and was stored in a sealed vessel. After sampling, the water and sediment samples were immediately delivered to the analytical laboratory. The bottom sediment samples were freeze-dried, passed through sieves, and then stored at −19 °C until analysis. To prepare bottom sediment samples, sieves with 0.6 mm and 0.25 mm gap widths were used with a WPS 210S moisture analyzer (RADWAG, Radom, Polska). Water samples were also collected from Lake Czechowice at a depth of 30 cm, into sterile PTFE bottles. The samples were analyzed on the day they were delivered to the laboratory. A Speedisk extraction kit (J.T. Baker, Deventer, The Netherlands) and octadecylsilane discs Bakerbond Speedisk^®^ C-18 (J.T. Baker) were used to separate analytes from water samples.

### 3.2. Preparation of Standard Solutions

Standard stock solutions (1 mg mL^−1^) of carbamazepine, ibuprofen, diclofenac, and acenocoumarol (IS) were prepared by dissolving the appropriate amount of standard in methanol. A series of working solutions was made by the serial dilution of standard stock solutions in methanol ranging from 0.5 to 100 µg mL^−1^. Quality control (QC) samples were prepared by the appropriate dilution of the working solution. Samples were tested at three concentration levels: low-quality control (LQC): 1.25 µg g^−1^ (for SUPRAS) and 0.5 μg mL^−1^ (for Speedisk), middle-quality control (MQC): 10.0 µg g^−1^ (for SUPRAS) and 2 μg mL^−1^ (for Speedisk), high-quality control (HQC): 20.0 µg g^−1^ (for SUPRAS) and 4 μg mL^−1^ (for Speedisk). All solutions were stored in the dark at 4 °C.

### 3.3. UHPLC-UV Conditions

A UHPLC system (Merck-Hitachi, Pliening, Germany) was used for separation carbamazepine, ibuprofen, diclofenac, and acenocoumarol. The UHPLC system included the following elements: a thermostatted column compartment (Model L-2350U), pump (Model L-2160U), autosampler (Model L-2200), absorbance detector (Model L-2400U), and a degasser module. A Poroshell 120 EC-C18 analytical column (100 mm × 3.0 mm, 2.7 µm, Agilent Technologies, Santa Clara, CA, USA) was used. The column temperature was set to 20 °C. The mobile phase consisted of a mixture of acetonitrile (solvent A) and 0.05% trifluoroacetic acid in water (TFA) (solvent B). The analytes were separated by gradient elution (Table 5). The injection volume was 3 μL, and the total run time of one analysis was 7.0 min. The internal standard was acenocoumarol. UV detection was performed at λ = 221 nm. The analyses were controlled using the EZ Chrom Elite program.

### 3.4. Preparation of Supramolecular Solvent and Determination of Its Volume

As part of the work, the influence of various factors, such as the THF:H_2_O (*v*/*v*) ratio and the amount of decanoic acid, on the composition and volume of the resulting supramolecular solvent was investigated. SUPRAS were prepared according to the procedure described below. The volume of the obtained supramolecular solvent was measured each time.

To 6.5 g of decanoic acid was added 70 mL of 10 mmol L^−1^ hydrochloric acid and 30 mL of THF. The mixture was stirred on a magnetic stirrer for 10 min and then separated into centrifuge tubes and centrifuged at 4000 rpm. After centrifuging, the upper organic supramolecular solvent layer was collected into a glass bottle, which was screwed and stored in a refrigerator at 4 °C. In this way, a supramolecular solvent with a composition of 30:70 (THF:H_2_O; *v*/*v*) was obtained. SUPRAS solvents with compositions of 25:75, 20:80, 15:85, 10:90, and 5:95 (THF:H_2_O; *v*/*v*) were prepared in a similar way. More THF was necessary to keep the liquid-liquid-phase separation at low temperature as a consequence of the decreased alkyl carboxylic acid solubility.

Solutions with different amounts of decanoic acid were also prepared to determine the optimal mass of DeA. For this purpose, 1, 3, 6.5, and 10 g of decanoic acid were weighed and quantitatively transferred to beakers. 70 mL of 10 mmol L^−1^ HCl and 30 mL of THF were added. The combination was mixed and centrifuged according to the procedure described above.

### 3.5. Preparation of Bottom Sediment Samples–SUPRAS Microextraction

The collected bottom sediment sample was dried in a mortar and sieved successively through sieves with 0.6 mm and 0.25 mm gap widths. The moisture content of the sample measured with a WPS 210S moisture analyzer was 2.5%. The collected bottom sediment was of sandy type and the organic carbon content was in the range of 0.4 to 3.1%. Bottom sediment samples were used to select SUPRAS microextraction conditions. For this purpose, 0.4 g of the bottom sediment sample was weighed and enriched so that the final concentration of drugs in the sample was 5 μg mL^−1^. The enrichment of the sediment sample was performed by adding a standard solution with a specific concentration dissolved in methanol (the entire surface of the sediment was covered), and then homogenized. The samples were evaporated to dryness and stored at 4 °C for 24 h to establish the adsorption equilibrium. A specific volume of supramolecular solvent was then added and mixed on a magnetic stirrer for a designated time. To accelerate the separation of solid particles from the liquid, the mixture was centrifuged at a certain rate for a designated time. The extract was collected and analyzed using UHPLC-UV.

The selection of SUPRAS microextraction conditions was based on literature data describing the optimization of SUPRAS microextraction procedures for other analytes (including herbicides, fungicides, dyes, ochratoxins, and PAH) and other real samples (including water, food, plant and animal tissues, soil) [22,23,24,25,26,27,28,29,57,58,59,60]. The volume ratio of THF:H_2_O, amount of DeA, the volume of supramolecular solvent, sample mass, extraction time, centrifugation time, and centrifugation speed were compared. The extraction procedure was verified based on the recovery and the enrichment factor (EF), defined as the ratio of the analyte concentration in the extract to the initial analyte concentration in the sample [61]:

(1) $$\mathrm{EF}=\frac{\mathrm{analyte}\;\mathrm{concentration}\;\mathrm{in}\;\mathrm{the}\;\mathrm{extract}\;\left[{µg\;\mathrm{mL}}^{-1}\right]}{\mathrm{analyte}\;\mathrm{concentration}\;\mathrm{in}\;\mathrm{the}\;\mathrm{sample}\left[{µg\;g}^{-1}\right]}$$

### 3.6. Preparation of Water Samples–Speedisk Technique

The analytes were isolated from water samples by solid-phase extraction using Bakerbond Speedisk^®^ C-18 extraction discs with octadecylsilane filling. Before extraction, water samples were acidified with sulfuric acid (VI) to pH = 4, after which known amounts of analytes were added. The sorbent was preconditioned with 15 mL of methanol followed by 20 mL of water. 500 mL of water was run through Speedisk using a vacuum. The Speedisk was dried for 30 min, and then elution was carried out with 15 mL of methanol. The eluate was evaporated to dryness. Finally, the residue was re-dissolved in 0.5 mL methanol, and 3 μL of the extract was injected into the chromatographic system. The extraction efficiency was determined by the recovery and enrichment factor (EF) calculated from Equation (2).

(2) $$\mathrm{EF}=\frac{\mathrm{analyte}\;\mathrm{concentration}\;\mathrm{in}\;\mathrm{the}\;\mathrm{extract}\;\left[{µg\;L}^{-1}\right]}{\mathrm{analyte}\;\mathrm{concentration}\;\mathrm{in}\;\mathrm{the}\;\mathrm{sample}\left[{µg\;L}^{-1}\right]}$$

### 3.7. Preparation of Blank Samples

Blank samples for SUPRAS microextraction were prepared by weighing 0.4 g of dried and sifted bottom sediment. Then, 700 μL supramolecular solvent (6.5 g DeA; THF:H_2_O = 30:70; *v*/*v*) was added, stirred on a magnetic stirrer for 20 min, and centrifuged at 9000 rpm for 10 min. The collected extract was used as a matrix to prepare the solutions used to obtain calibration curves. Seven blank samples were made in the same way.

Blank samples for the Speedisk technique were made by using sulfuric acid (VI) to acidify 500 mL of water to pH = 4–5. Solid-phase extraction was then carried out using the Speedisk technique, the procedure for which is described above. The eluate was evaporated to dryness, and the residue was dissolved in 0.5 mL methanol. Seven blank samples (water extracts) were prepared.

### 3.8. Method Validation

The newly-developed UHPLC-UV method, used to separate and determine DIC, IBU, and CBZ in bottom sediment and water samples, was validated in terms of its limit of detection (LOD), limit of quantification (LOQ), linearity, accuracy, precision, and recovery.

The linearity of the developed UHPLC-UV method was determined based on standard curves prepared during three independent runs. Calibration curves of DIC, IBU, and CBZ were prepared in the range from 0.5 to 25 μg g^−1^ for SUPRAS microextraction and 0.05 to 5 μg L^−1^ for the Speedisk technique. An internal standard was added each time, the final concentration of which was 10 μg g^−1^ for SUPRAS microextraction and 2 μg L^−1^ for the Speedisk technique. A regression analysis of the obtained data was performed, and then the coefficient of determination (R^2^) was calculated for each compound.

The lowest analyte concentration for which the relative error was less than 20% was taken as the limit of quantification (LOQ). The limit of detection (LOD) was calculated based on Equation (3):

(3) $$\mathrm{LOD}=\frac{\mathrm{LOQ}}{3}$$

To determine accuracy and precision, QC samples were prepared at low, middle, and high-concentration levels according to the procedure described above. For each QC sample, six replicates were performed. Based on the obtained results, the mean concentration of a drug was calculated, then the accuracy was determined using the relative error (RE) (Equation (4)) and precision, which were measured by the relative standard deviation (RSD) (Equation (5)) between the replicate measurements expressed as a percent coefficient of variation (%CV) (Equation (6)):

(4) $$\mathrm{RE}\;\left(\%\right)=\left(\frac{\mathrm{measure}\;\mathrm{value}-\mathrm{theoretical}\;\mathrm{value}}{\mathrm{theoretical}\;\mathrm{value}}\right)\cdot 100\%$$

(5) $$\mathrm{RSD}=\frac{\sqrt{\frac{\sum_{i=1}^{n}{\left(\mathrm{single}\;\mathrm{measurement}\;\mathrm{concentration}\;-\;\mathrm{mean}\;\mathrm{concentration}\right)}^{2}}{n}}}{\mathrm{mean}\;\mathrm{concentration}}$$

(6) CV (%)=RSD·100%

The extraction procedures were verified based on extract recovery using the SUPRAS-UHPLC-UV method. The recovery was calculated as the ratio of the measured concentration of the compound in the sample (measured based on the area under the peak) to the concentration of the compound if the extraction was 100% efficient:

(7) $$\mathrm{Recovery}\;\left(\%\right)=\frac{\mathrm{measure}\;\mathrm{value}}{\mathrm{theoretical}\;\mathrm{value}}\cdot 100\%$$

### 3.9. Study of Sorption Efficiency of Selected Drugs on Sediment

The efficiency of the sorption of tested drugs on sediment was investigated. To a 500 mL flat-bottom flask was added 3 g of sediment and 30 mL of deionized water, previously enriched with known amounts of CBZ, DIC, and IBU. Three replicates were performed at eight concentration levels in the range of 80–1500 μg L^−1^. The solutions were shaken for 24 h, decanted, and centrifuged for 10 min at 6500 rpm, followed by double analysis using UHPLC. The results obtained were compared with the results obtained for working solutions of the same concentrations to determine the amount of compound that had been sorbed. The adsorption efficiency was investigated according to the procedure described in ref. [62].

## 4. Conclusions

In this study, a novel supramolecular solvent (SUPRAS)-based microextraction method was developed to isolate selected anticonvulsants and NSAIDs from sediment samples. The proposed SUPRAS supported green sample preparation by using a surfactant (nontoxic agent) as the extractant, and the extraction was performed at ambient temperature with a short extraction time. The Speedisk method was used to isolate selected pharmaceuticals from water samples. The extraction procedure was developed and optimized, and a high recovery of all analytes was obtained. The recovery using SUPRAS extraction was 88.5–115%, and for Speedisk extraction, it was 81.0–106%. Overall, the proposed SUPRAS-based microextraction is attractive and can be used as an alternative extraction method with off-line preconcentration. A new UHPLC-UV method was also developed for the chromatographic separation of selected drugs. For the first time, a column obtained in the core-shell technology to determine drugs in sediment or soil samples was used. The use of this technology allows for much higher sensitivity and efficiency than when using other columns. The method has been validated, and validation parameters such as recovery, sensitivity, linearity, accuracy, and precision were satisfactory. The determined detection limit of the analytes using SUPRAS microextraction was 0.42 μg g^−1^ for bottom sediment, and for the Speedisk extraction, it was 0.5 μg L^−1^ from water. As part of the work, the effectiveness of the sorption of selected drugs on soil was also determined. Only ibuprofen was adsorbed, which stemmed from the assumed specific properties of the soil sample and the pH favouring ibuprofen adsorption. A new safer, environmentally-friendly, and faster extraction method for selected pharmaceuticals was developed in this study, which can be successfully applied to environmental matrices.

## Figures and Tables

**Table 1:** Drug recovery after applying the developed SUPRAS procedure and Speedisk solid phase extraction procedure (*n* = 6).

Compound	Concentration (µg g^−1^)/(µg L^−1^)	Recovery (%)	RSD (%)
SUPRAS procedure			
CBZ	1.25	115	7.2
	10	96.3	4.1
	20	98.8	7.3
DIC	1.25	97.0	8.2
	10	88.5	3.5
	20	90.8	1.6
IBU	1.25	112	2.8
	10	103	0.3
	20	98.4	3.2
Speedisk solid phase extraction procedure			
CBZ	0.5	100	1.1
	2	97.8	2.4
	4	97.3	0.6
DIC	0.5	103	2.6
	2	106	2.0
	4	103	1.8
IBU	0.5	81.0	2.9
	2	98.5	1.8
	4	102	5.6

**Table 2:** Linear regression equations of calibration curves and calculated regression parameters (*n* = 6).

Compound	Linear Range	Calibration Curve Equation	Sxy	Sa	Sb	R^2 a^	LOQ ^b^	LOD ^c^
**Bottom sediment samples**								
**CBZ**	0.5–25.0 µg g^−1^	y = 0.4853x + 0.1092	0.4635	0.0204	0.2619	0.9912	1.25 µg g^−1^	0.42 µg g^−1^
**DIC**		y = 0.1196x + 0.0517	0.0605	0.0027	0.0342	0.9975		
**IBU**		y = 0.0786x + 0.0849	0.0413	0.0018	0.0233	0.9973		
**Water samples**								
**CBZ**	0.05–5.0 µg L^−1^	y = 0.6159x − 0.0264	0.0191	0.0042	0.0118	0.9998	0.05 µg L^−1^	0.017 µg L^−1^
**DIC**		y = 0.1571x − 0.0004	0.0132	0.0029	0.0081	0.9983		
**IBU**		y = 0.2503x + 0.0217	0.0317	0.0070	0.0196	0.9961		

^a^ R^2^—correlation coefficient.; ^b^ LOD—limit of detection.; ^c^ LOQ—limit of quantification.

**Table 3:** Comparison of the developed SUPRAS microextraction method with other methods to extract drugs from sediment samples described in the literature.

Method	Detection	Compound	LOD/LOQ	Linear Range	Recovery (%)	Volume of Extractant	Extraction/Analysis Time	**Ref.**
PLE/SPE-LC	MS/MS	ibuprofen diclofenac carbamazepine	LOD 3.0–30.0 ng g^−1^	0.05–10 µg g^−1^	35–135	1 mL	11 min + SPE/45 min	[9]
SLE/SPE-UPLC	MS/MS	ibuprofen diclofenac	-	–	85–106.5	1 mL	210 min + SPE/–	[10]
PLE/SPE-HPLC	MS/MS	diclofenac carbamazepine	LOQ 0.2–2.0 ng g^−1^	–	–	1 mL	15 min + SPE/–	[11]
PLE-HPLC	MS	ibuprofen diclofenac carbamazepine	LOQ 14.0–29.0 ng g^−1^	10–100 µg L^−1^	68–112	40 mL	40 min/25–30 min	[12]
USE-SPE-HPLC	DAD	ibuprofen diclofenac carbamazepine	LOQ 1.1–187.0 ng g^−1^	–	85.8–102	150 µL	35.5 min + SPE/–	[13]
PLE-SPE-LC USE-SPE-LC	MS/MS	ibuprofen diclofenac carbamazepine	-	–	40–130	1 mL	15–35 min + SPE/–	[14]
USE-SPE-LC	MS/MS	ibuprofen diclofenac carbamazepine	LOQ 20.0 ng g^−1^	10 ng g^−1^–20 µg g^−1^	44–81	200 µL	10 min + SPE/28 min	[15]
PLE-SPE-LC	MS/MS	ibuprofen dikolfenak carbamazepine	MQL 0.78–163.7 ng g^−1^	10 ng g^−1^–2 µg g^−1^	60–82	1 mL	31 min + SPE/–	[16]
SBSE-HF-LPME-LC	MS	ibuprofen diclofenac	-	0.5–8 mg L^−1^	57–62	10 µL	20–22 h/–	[17]
PLE-HF-LPME-LC	MS	ibuprofen diclofenac	MLD 0.4–1.4 ng g^−1^	3.9–4000 ng mL^−1^	101–109	25 µL	176 min/26 min	[18]
SUPRAS-UHPLC	UV	ibuprofen diclofenac carbamazepine	LOD 0.42 µg g^−1^	0.5–25 µg g^−1^	88.5–115	700 µL	30 min/7 min	this work

**Table 4:** Comparison of the developed Speedisk solid phase extraction method with other methods to extract drugs from water samples described in the literature.

Method	Detection	Compound	LOD/LOQ	Linear Range	Recovery (%)	Volume of Extractant	Extraction/Analysis Time	Ref.
SPE-GC	MS	ibuprofen diclofenac carbamazepine	LOD: 12–32 ng L^−1^	5–50 ng L^−1^	67–80	200 µL	SPE/62 min	[35]
SPE-GC	MS	ibuprofen diclofenac carbamazepine	LOQ: 0.07–0.09 ng L^−1^	–	70–100	50 µL	SPE/38 min	[36]
SPE-LC	MS	ibuprofen diclofenac carbamazepine	–	–	46–97	2 mL	SPE/40 min	[37]
SPE-LC	MS/MS	ibuprofen diclofenac	LOD: 20 ng L^−1^	–	62–117	–	–	[38]
SPE-GC	MS	ibuprofen diclofenac carbamazepine	LOQ: 2–8 ng L^−1^	0.1–10 ng µL^−1^	77–93	100 µL	SPE/32 min	[39]
SPE-GC	MS	ibuprofen diclofenac	LOD: 36–38 ng L^−1^	10–2000 ng L^−1^	67–76	100 µL	SPE/50.5 min	[40]
SPE-HPLC	MS/MS	ibuprofen diclofenac	LOD: 0.14–0.52 µg L^−1^	1–250 µg L^−1^	56–77	300 µL	SPE/20–43 min	[41]
SPE-GC	MS	ibuprofen dikolfenak carbamazepine	LOQ: 5–10 ng L^−1^	10 ng g^−1^–2 µg g^−1^	50–99	0.5 mL	SPE/–	[42]
SPE-GC	MS	ibuprofen diclofenac	ILD: 20–32 µg L^−1^	0.02–25 mg mL^−1^	84–157	130 µL	SPE/56 min	[43]
SPE-GC	MS	ibuprofen diclofenac carbamazepine	LOD: 0.01–0.02 ng L^−1^	0.06–400 ng L^−1^	92–102	105 µL	SPE/–	[44]
Speedisk-UHPLC	UV	ibuprofen diclofenac carbamazepine	LOD: 0.17 µg L^−1^	0.05–10 µg L^−1^	81–106	500 µL	60 min/7 min	this work

**Table 5:** The gradient elution program and the mobile phase flow rate.

Time (min)	Solvent A (%) ^a^	Solvent B (%) ^a^	Flow Rate (mL min^−1^)
0.0	40	60	0.2
1.0	45	55	0.7
2.0	45	55	0.6
3.0	45	55	0.5
3.5	80	20	0.7
4.0	80	20	0.7
4.5	85	15	0.4
4.9	85	15	0.3
5.0	100	0	0.7
7.0	100	0	0.7

^a^ Solvent A: acetonitrile, solvent B: 0.05% TFA in water.

## Supplementary Materials

The following are available online. Table S1. Properties of selected drugs. Table S2. Accuracy and precision of the developed methods. Figure S1. Effect of the amount of DeA on the recovery of analytes. Figure S2. Influence of supramolecular solvent volume on recovery and enriching analytes. Figure S3. The effect of sample mass on recovery and enrichment of analytes. Figure S4. Influence of extraction time on recovery of analytes. Figure S5a. Influence of centrifugation time on recovery of analytes. Figure S5b. he effect of centrifugation rotation on the recovery of analytes. Figure S6. Freundlich adsorption isotherm for ibuprofen. Figure S7. Determination of the *K*_D_ parameter for ibuprofen.
