# Supplementary material for: Supramolecular Solvent-Based Microextraction of Selected Anticonvulsant and Nonsteroidal Anti-Inflammatory Drugs from Sediment Samples
Source: Molecules. 2020 Dec 1;25(23):5671. doi: 10.3390/molecules25235671 (PMC7729498; doi:10.3390/molecules25235671)
Supplement: Supplementary file 1 [file molecules-25-05671-s001.pdf]

Supramolecular solvent-based microextraction of selected anticonvulsant and nonsteroidal anti-inflammatory drugs from sediment samples

Sylwia Bajkacz[1,*], Paulina Adamczewska[1], Klaudia Kokoszka[1],
Elżbieta Kycia-Słocka[1], Adam Sochacki[2,3], Ewa Felis[2]

[1] Silesian University of Technology, Faculty of Chemistry, Department of Inorganic Chemistry, Analytical Chemistry and Electrochemistry, B. Krzywoustego 6 St., 44-100 Gliwice, Poland
[2] Silesian University of Technology, Faculty of Power and Environmental Engineering, Environmental Biotechnology Department, Akademicka 2 St., 44-100 Gliwice, Poland
[3]Czech University of Life Sciences Prague, Faculty of Environmental Sciences, Department of Applied Ecology, Kamýcká 129, 165 00 Prague, Czech Republic

SUPPLEMENTARY INFORMATION

Summary

This supporting information file includes additional results and information as described in the text of the main article including:

Table S1. Properties of selected drugs.
Table S2. Accuracy and precision of the developed methods.
Figure S1. Effect of the amount of DeA on the recovery of analytes.
Figure S2. Influence of supramolecular solvent volume on recovery and enriching analytes.
Figure S3. The effect of sample mass on recovery and enrichment of analytes.
Figure S4. Influence of extraction time on recovery of analytes.
Figure S5a. Influence of centrifugation time on recovery of analytes.
Figure S5b. The effect of centrifugation rotation on the recovery of analytes.
Figure S6. Freundlich adsorption isotherm for ibuprofen.
Figure S7. Determination of the K_D parameter for ibuprofen.

[*] Corresponding author.
E-mail address: sylwia.bajkacz@polsl.pl (S. Bajkacz).

Table S1. Properties of selected drugs.

Compound	***Structural formula***	***logP***	***pK$_a$***
**CBZ**	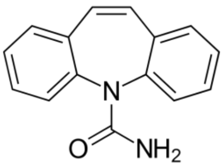	2.77	13.9
**ACE**	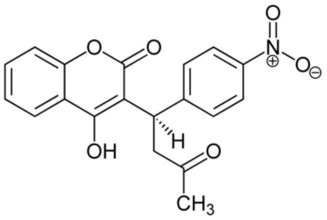	3.42	5.1
**DIC**	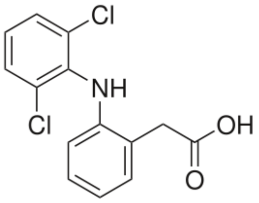	4.26	4.1
**IBU**	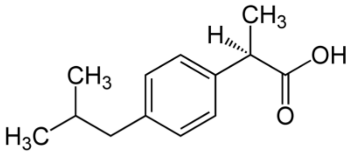	3.94	4.9

Table S2. Accuracy and precision of the developed methods.

Compound	*Theoretical concentration (μg g^{-1}) / (μg L^{-1})*	*Measure concentration (μg g^{-1}) / (μg L^{-1})*	*SD[a] (μg g^{-1}) / (μg L^{-1})*	*RE[b] (%)*	*CV[c] (%)*
Bottom sediment samples					
	1.25	1.41	0.062	13	10
**CBZ**	10	11.1	0.44	11	9.1
	20	20.9	1.32	4.9	12
	1.25	1.32	0.061	5.5	11
**DIC**	10	10.8	0.54	7.8	11
	20	19.8	1.13	-0.8	11
	1.25	1.23	0.032	-1.5	5.8
**IBU**	10	10.8	0.24	8.2	5.4
	20	19.3	1.14	-3.5	11
Water samples					
	0.5	0.48	0.042	-4.9	9.2
**CBZ**	2	2.1	0.13	4.9	3.4
	4	4.1	0.34	1.4	6.9
	0.5	0.45	0.034	-10	7.7
**DIC**	2	2.1	0.003	3.8	0.9
	4	4.1	0.35	7.5	6.4
	0.5	0.47	0.044	-6.0	8.9
**IBU**	2	2.2	0.004	11	0.6
	4	4.1	0.32	2.1	7.1

[a] SD – standard deviation
[b] RE – relative error
[c] CV – coefficient of variation

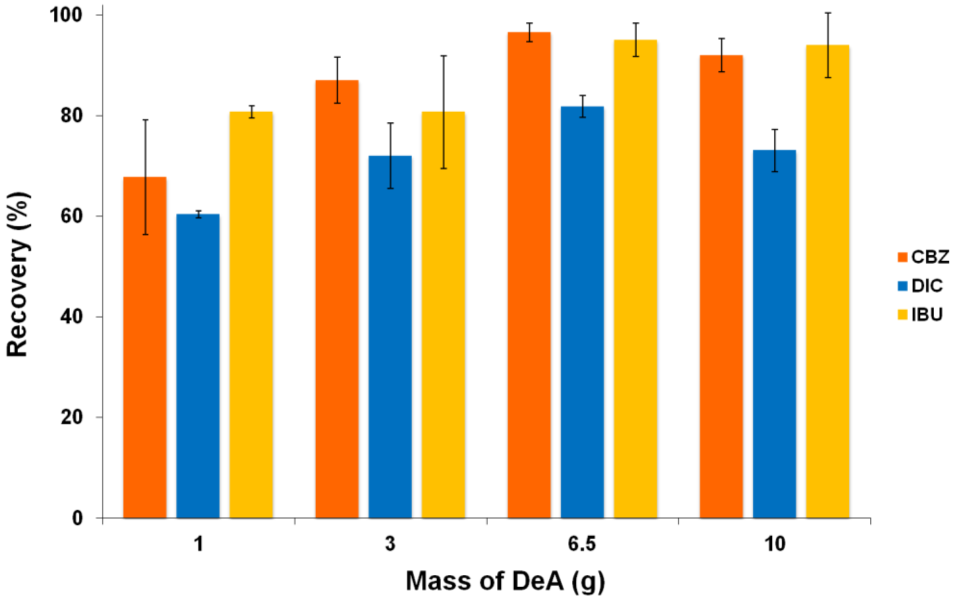

Figure S1. Effect of the amount of DeA on the recovery of analytes.

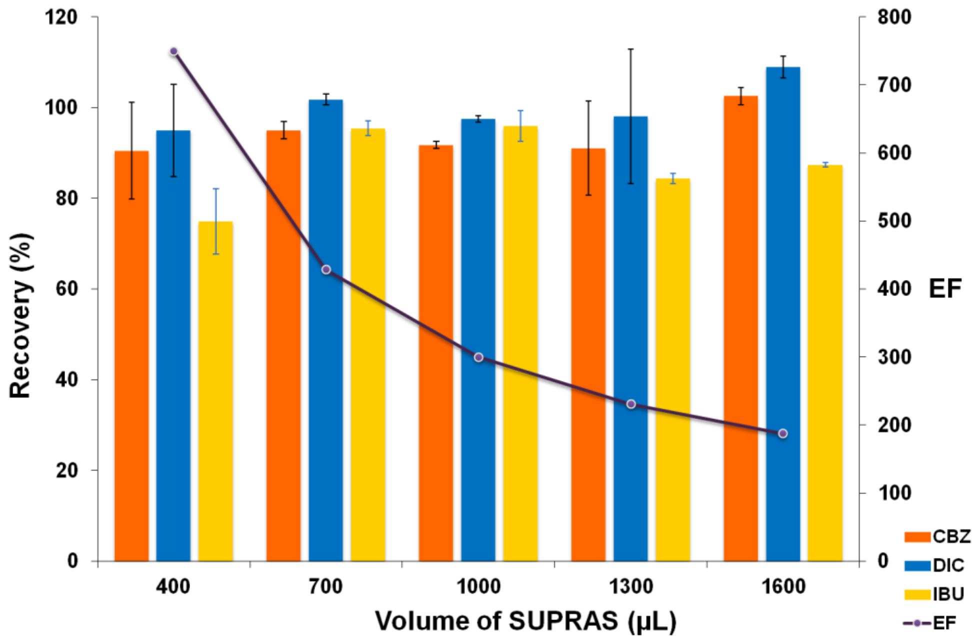

Figure S2. Influence of supramolecular solvent volume on recovery and enriching analytes.

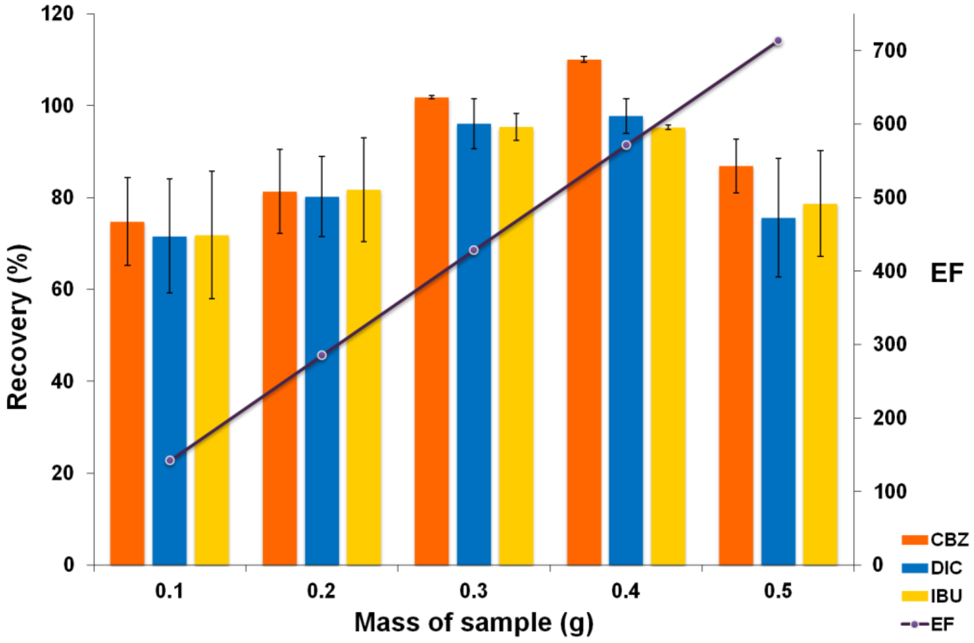

Figure S3. The effect of sample mass on recovery and enrichment of analytes.

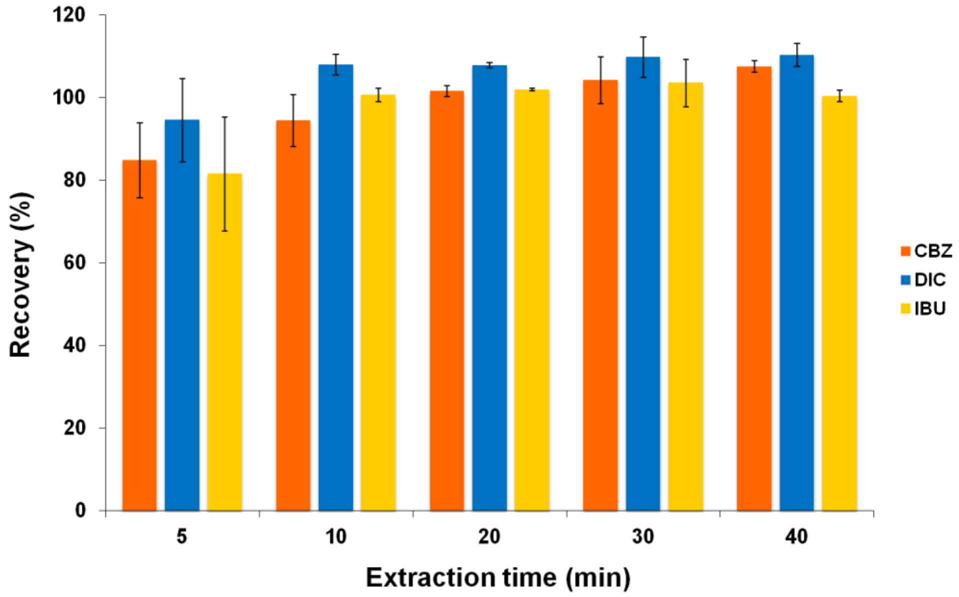

Figure S4. Influence of extraction time on recovery of analytes.

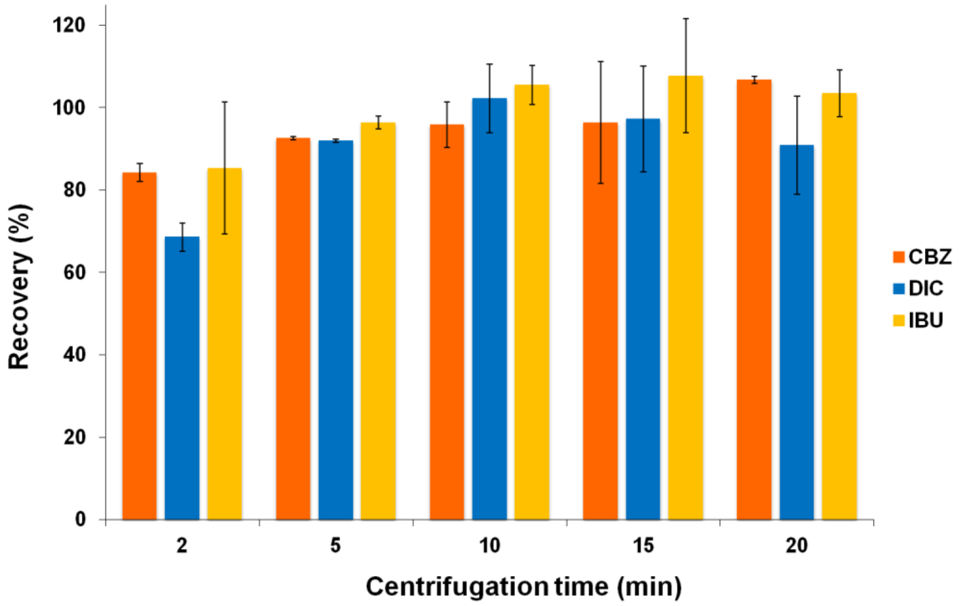

Figure S5a. Influence of centrifugation time on recovery of analytes.

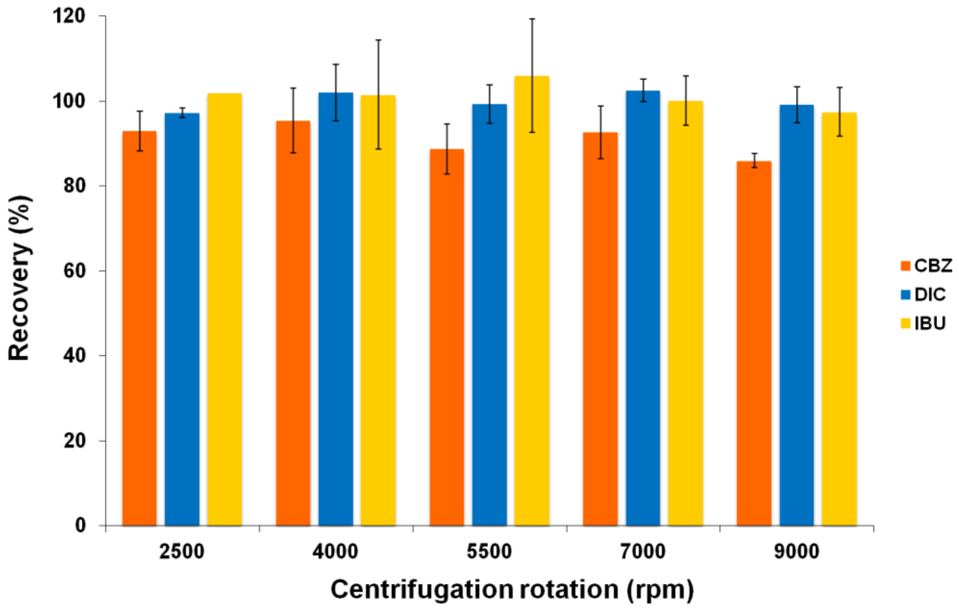

Figure S5b. The effect of centrifugation rotation on the recovery of analytes.

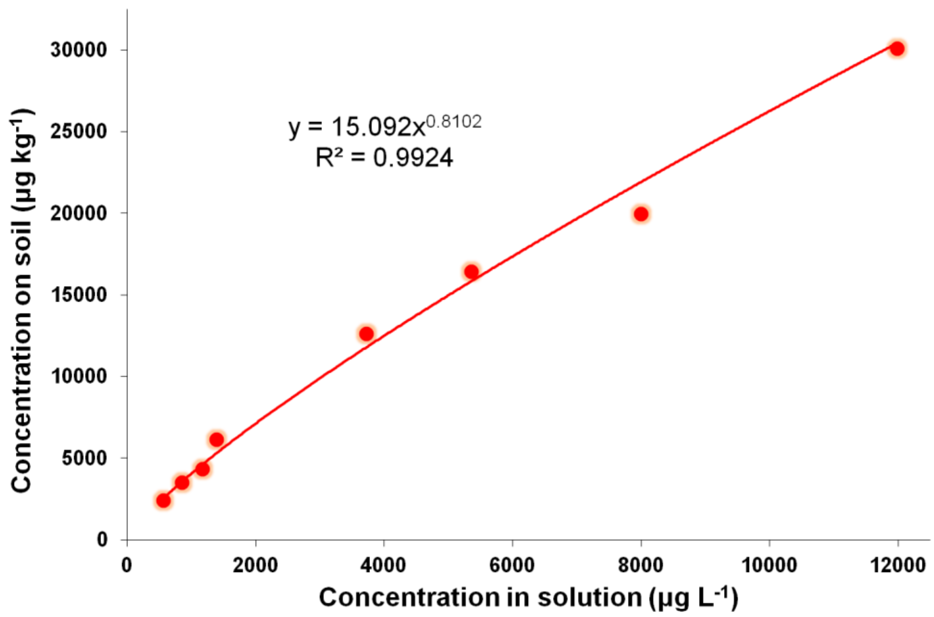

Figure S6. Freundlich adsorption isotherm for ibuprofen.

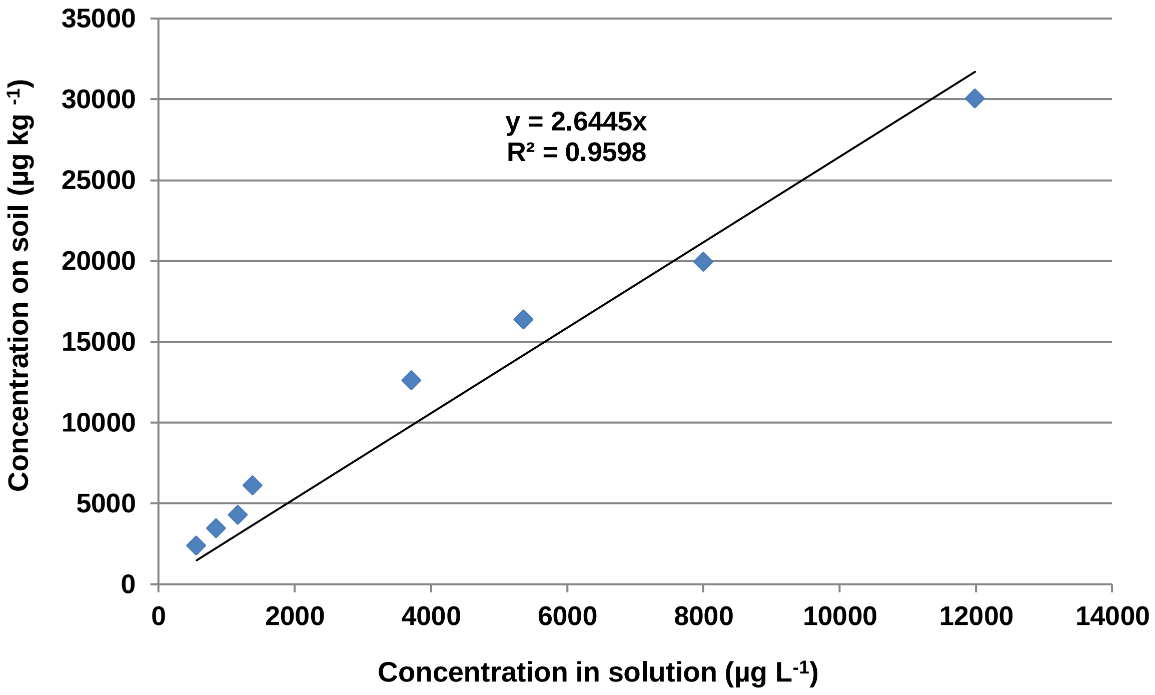

Figure S7. Determination of the K_D parameter for ibuprofen.
